# Recovery from eating disorders: a guide for clinicians and their clients

**DOI:** 10.1186/2050-2974-2-12

**Published:** 2014-06-02

**Authors:** Stephen Touyz

**Affiliations:** 1 School of Psychology, University of Sydney, Mackie Building (K01), NSW 2006, Australia

**Keywords:** Anorexia nervosa, Recovery, Eating disorders

## Book details

Recovery from eating disorders: a guide for clinicians and their clients, Greta Noordenbos, Wiley-Blackwell 2013.

## Review

This is a short affordable softcover guide book for families, carers and patients. As Professor Bob Palmer so elegantly points out on the cover of this guidebook, it will be an excellent resource. The road to recovery is often a difficult and challenging one with what appear to be insurmountable obstacles to overcome. This journey will now be considerably easier with both the knowledge and support found in this guidebook.

This book is written in a warm and friendly manner and is easy to read. It provides a wealth of clinical information often in the form of clinical vignettes and practical exercises to aid recovery for patients and/or families. The message throughout is one of hope and inspiration despite the innumerable odds faced by so many of these patients. It is truly a comprehensive guide with a focus not only on existing eating disorders but on which early warming signs to look out far as well.

Greta Noordenbos is one of the few experts in the field of eating disorders who has a special clinical/research interest in recovery. Her rich knowledge and clinical experience brings this book alive. She is at pains to point out that recovery is much more than normalizing eating behavior and weight as well as ceasing to binge or purge. She provides excellent commentary on the importance of a positive body attitude, improving self esteem, expressing emotions and improving interpersonal relationships. Other important topics such as extreme dieting, the negative consequences of eating disorders, the turning point and motivation to recovery normalizing eating habits and physical recovery are also given due diligence.

Those reading the book will never be short of inspiration and hope in their own journeys. Clinicians with a special interest in recovery will also find the insights gained very useful to use in their own clinical sessions.

Professor Stephen Touyz

## Competing interests

Co-editor journal of eating disorders.

